# Boiling and Frying Peanuts Decreases Soluble Peanut (*Arachis Hypogaea*) Allergens Ara h 1 and Ara h 2 But Does Not Generate Hypoallergenic Peanuts

**DOI:** 10.1371/journal.pone.0157849

**Published:** 2016-06-16

**Authors:** Sarah S. Comstock, Soheila J. Maleki, Suzanne S. Teuber

**Affiliations:** 1 Department of Internal Medicine, Division of Rheumatology, Allergy and Clinical Immunology, School of Medicine, University of California Davis, Davis, California, United States of America; 2 United States Department of Agriculture-Agricultural Research Service-Southern Regional Research Center (USDA-ARS-SRRC), New Orleans, Louisiana, United States of America; INRA, FRANCE

## Abstract

Peanut allergy continues to be a problem in most developed countries of the world. We sought a processing method that would alter allergenic peanut proteins, such that allergen recognition by IgE from allergic individuals would be significantly reduced or eliminated. Such a method would render accidental exposures to trace amounts of peanuts safer. A combination of boiling and frying decreased recovery of Ara h 1 and Ara h 2 at their expected MWs. In contrast, treatment with high pressures under varying temperatures had no effect on protein extraction profiles. Antibodies specific for Ara h 1, Ara h 2, and Ara h 6 bound proteins extracted from raw samples but not in boiled/fried samples. However, pre-incubation of serum with boiled/fried extract removed most raw peanut-reactive IgE from solution, including IgE directed to Ara h 1 and 2. Thus, this method of processing is unlikely to generate a peanut product tolerated by peanut allergic patients. Importantly, variability in individual patients’ IgE repertoires may mean that some patients’ IgE would bind fewer polypeptides in the sequentially processed seed.

## Introduction

Peanut allergy continues to be a problem in most developed countries of the world, particularly in the United States where peanuts and peanut products are commonly consumed. To date, although clinical trials of oral immunotherapy [1] and several other approaches, such as early introduction of peanut (LEAP study [2]), are showing promise, peanut allergic individuals still must carefully avoid exposure to peanuts. A processing method which would raise the quantitative oral threshold (around 1.6mg for peanut [3], with minimal eliciting doses of peanut estimated to be 0.14mg for children and 0.21mg for adults [4]) for an objective allergic reaction by any degree would be beneficial to peanut growers, food processors and peanut-allergic individuals alike. Such a processing method would increase the safety of the food supply by making accidental contamination less harmful for individuals with severe peanut allergy.

Peanuts contain between 23% and 27% protein. Major peanut allergens include Ara h 1 (conarachin, 7S globulin, vicilin) [5], Ara h 2 (2S albumin) [6] and Ara h 3 (glycinin, 11S storage protein) [7]. Other peanut allergens include Ara h 5 (profilin) [8], Ara h 6 (2S albumin) [9,10], Ara h 7 (2S albumin) [9], Ara h 8 (Bet v 1-related) [11,12], Ara h 9 (lipid transfer protein) [13,14], Ara h 10/11(oleosins) [15–17], and Ara h 12/13 (defensins) [18], among others (for a full list see the WHO/IUIS Allergen Database at www.allergen.org). In a quantitative analysis of peanuts, Ara h 1 accounted for between 12% and 16% of total protein, and Ara h 2 accounted for 5.9% to 9.3% of total peanut protein content [19]. Peanut allergens are generally stable proteins under ambient and digestive conditions.

A processing method with the potential to decrease IgE-reactivity has been previously sought [20–30]. Paradoxically, it has been shown that standard roasting of peanuts actually increases IgE binding to Ara h 1 and Ara h 2 [22,26,31]. However, fewer studies have looked at combinations of processing methods to alter the allergenicity of foods [23,29,30,32]. Because frying and boiling each had been shown to decrease the presence of highly allergenic peanut proteins in peanut extracts [20,27,33], and high heat [32] and high pressure [24] had been shown to decrease allergenicity of peanut allergens, we characterized the IgE binding capabilities of protein extracts from peanuts that were untreated (raw), or treated by a boiling and frying process (boiled/fried) and then subjected to various pressure/temperature/time treatments. To determine if the allergens were destroyed, rendered insoluble or altered such that they migrated at an unexpected MW, immunoblotting experiments were undertaken.

## Materials and Methods

### Peanut samples

Peanut pastes from either *Arachis hypogaea cv*. Runner type A (United States) or type B (China) were provided by Masterfoods Inc. in vacuum packed sachets. These pastes were made from peanuts that had been (1) boiled in a water bath 5 minutes and then pan fried in oil at 175°C (boiled/fried) for 130 seconds or (2) untreated (raw). After heating, all peanuts were subsequently exposed to conditions of varying temperatures and pressures for varying amounts of time (Table 1) prior to pulverizing into a paste.

**Table 1:** Peanut Samples.

Assigned ID Number	Treatment	Pressure (mPa)	Temperature (°C) During Pressure Treatment	Time (minutes) of Pressure Treatment
1	Boiled/Fried	200	20	5
2	Raw	200	20	5
3	Boiled/Fried	200	80	5
4	Raw	200	80	5
5	Boiled/Fried	600	50	20
6	Raw	600	50	20
7	Boiled/Fried	600	80	20
8	Raw	600	80	20
9	Boiled/Fried	200	20	20
10	Raw	200	20	20
11	Boiled/Fried	200	50	20
12	Raw	200	50	20
13	Boiled/Fried	600	20	5
14	Raw	600	20	5
15	Boiled/Fried	400	50	5
16	Raw	400	50	5
17	Boiled/Fried	600	20	20
18	Raw	600	20	20
19	Boiled/Fried	400	20	5
20	Raw	400	20	5
21	Boiled/Fried	400	50	20
22	Raw	400	50	20
23	Boiled/Fried	400	80	5
24	Raw	400	80	5
25	Boiled/Fried	600	80	5
26	Raw	600	80	5
27	Boiled/Fried	400	20	20
28	Raw	400	20	20
29	Boiled/Fried	200	50	5
30	Raw	200	50	5
31	Boiled/Fried	200	80	20
32	Raw	200	80	20
33	Boiled/Fried	400	80	20
34	Raw	400	80	20
35	Boiled/Fried	600	50	5
36	Raw	600	50	5
37	Boiled/Fried	Control	0	0
38	Raw	Control	0	0

### Human sera

Sera from one control and seven patients known to have IgE to the major peanut allergens were used. All participants were adults (>20 years old) who gave written informed consent, and the use of human serum for these *in vitro* studies of food allergy was approved by the University of California, Davis, Institutional Review Board. Serum from patients with a clear history of clinical reactions to peanuts and confirmed peanut specific IgE (>0.35kU/L (Phadia AB, Uppsala, Sweden) and/or positive immunoblot) were used (Table 2). Serum from an atopic (mold sensitive), non-food allergic patient was used as a control (subject 1). With the exception of the control (subject 1) and one of the peanut-allergic (subject 6) participants, all subjects had experienced a life-threatening reaction to consumption of peanut that resulted in an emergency room visit.

**Table 2:** Subject Peanut Specific-IgE.

Subject ID	Peanut ImmunoCAP (kU/L of serum)
1	Control: atopic (mold sensitive), non-food allergic patient
2	>100
3	20.4
4	>100
5	21.2
6	96.6
7	1.59
8	7.2

### Defatting procedures

For chloroform/methanol (CM) defatting, 1g of peanut paste was incubated with 10ml CM (2 parts C to 1 part M) at 4°C for 15min with gentle agitation. Solutions were centrifuged at 2800rpm for 10min at 4°C in a Sorvall RC3B swinging bucket rotor. The supernatant was disposed of and 10ml of CM was added to the pellet. The above procedures were repeated until supernatants looked clear, about three times. After the final centrifugation, the supernatant was removed and samples were tapped out into large weigh boats to sit in a chemical fume hood overnight (ON) to ensure all CM was evaporated from the samples. Peanut protein was extracted from these powders the following morning.

### Peanut extractions

Peanut samples were extracted with Buffer D (50mM Tris-HCl, pH 8.0, 22% v/v glycerol, 1% w/v polyethylene glycol 8000, 7mM citric acid, 6mM L-cysteine, 6mM L-ascorbic acid, 2mM EDTA, 4% w/v polyvinyl polypyrrolidone (PVPP)). Two grams of peanut paste or defatted peanut powder were incubated in 10ml Buffer D ON at 4°C with gentle agitation. Solutions were centrifuged at 14000 x *g* for 30min at 4°C. Supernatants were filtered through 5μm syringe filters and stored as 1ml aliquots at -70°C. Protein concentrations were determined using the Coomassie Plus Protein Assay Reagent Kit (Pierce, Rockford, IL).

### SDS-PAGE

Samples were run on 12 or 14% SDS-PAGE gels under either reducing or non-reducing conditions [34] as indicated. The amount of protein loaded varied and is indicated in each figure. Gels were either transferred to nitrocellulose (NC) or stained with Coomassie Brilliant Blue (CBB) protein stain.

### Protein transfer and immunoblotting

Proteins were transferred to 0.22μm NC at a constant 30V ON at room temperature (RT). Membranes were Ponceau S stained to ensure proper protein transfer and destained before immunoblotting. NC was used as one large block or cut into 3mm x 10cm strips and incubated for 1 hr in block (5% nonfat dry milk/PBS/0.1% Tween 20) on a rocking platform at RT. Strips were then incubated with human sera diluted in block at 4°C ON with gentle rocking. After one 15min wash in wash buffer (PBS/0.1% Tween 20) and three 5min washes, secondary antibody, HRP-labeled mouse anti-human IgE (Southern Biotechnologies, Birmingham, AL) diluted 1:6000 in block was added to the strips. Secondary incubation was at RT for 105min. Membranes were washed as before. Each strip was incubated with 600μl and each full membrane was incubated with 10ml chemiluminescent reagent (Supersignal West Pico, Pierce Biotechnologies, Rockford, IL) at RT for 4min on a rocker platform and exposed to film (Phenix FBX-810) ON.

### Dot blotting

Using a lead pencil, a grid was drawn on the NC. Varying μg amounts of protein were dotted in each square, typically 1μg per spot. Dots were allowed to dry. NC was rinsed with wash buffer and put in block (Sigma block, Sigma B-6429) for 1 hr at RT with rocking. After blocking, sera diluted in block, as indicated in each figure, was added to the NC and incubated with the membrane ON at RT on a rocking platform. After one 15min wash and three 5min washes, secondary antibody, HRP-labeled mouse anti-human IgE (Southern Biotechnologies) diluted 1:6000 in block was added to the NC. The secondary was incubated with the NC for 105min at RT on a rocking platform. The previous washing regimen was repeated and chemiluminescent reagents were used as indicated above.

### Immunoblotting with peanut allergen-specific antibodies

Proteins were either transferred ON at a constant 30V or dotted onto 0.22μm NC. Membranes were Ponceau S stained to ensure proper protein transfer and destained before immunoblotting. NC was kept as a full membrane or cut into 3mm x 10cm strips and incubated for 1hr in blocking buffer (Sigma block) at RT with gentle rocking. Primary antibodies against peanut allergens were obtained from chickens (made by Sigma Immunosys, The Woodlands, TX). These polyclonal antibodies have been shown to recognize allergens in raw and processed peanut samples [35,36]. Anti-Ara h 1 (1:5000), anti-Ara h 2 (1:8000), anti-Ara h 3 (1:5000) or anti-Ara h 6 (1:5000), were diluted in blocking buffer. Membranes were incubated with diluted primary antibody for 1hr at RT with gentle rocking. After one 15min wash in wash buffer (PBS/0.1% Tween 20) and three 5min washes, secondary antibody, HRP-labeled goat anti-chicken IgY (Gallus Immunotech, Cary, NC), was added to the strips. The secondary was diluted 1:50,000 in blocking buffer. Secondary incubation was at RT for 30min with gentle rocking. Strips were then washed following the previous regimen. Strips were incubated with chemiluminescent substrate (SuperSignal West Pico, Pierce Biotechnologies, Rockford, IL) at RT for 4min with gentle rocking and exposed to film (Phenix FBX-810) ON.

### Inhibition immunoblotting

Sera, diluted 1:10 in blocking buffer (PBS/5% milk/0.01% Tween 20), were incubated ON at 4°C with boiled/fried peanut extract (25μg, 50μg, or 100μg in a 500μL reaction volume), purified Ara h 2 (5μg, 10μg/mL), purified Ara h 6 (5μg, 10μg/mL), or perennial ryegrass pollen extract (200μg/mL) and then used in immunoblotting as above. Absorptions with perennial ryegrass pollen extract and diluent alone were negative controls. Pollen extract from *L*. *perenne* desiccated pollen (Hollister-Stier, Spokane, WA) was prepared as previously described [37]. Purified peanut allergens were prepared as previously described [38].

## Results

### Defatting does not affect the protein profile of peanut extracts

**Fig 1:**
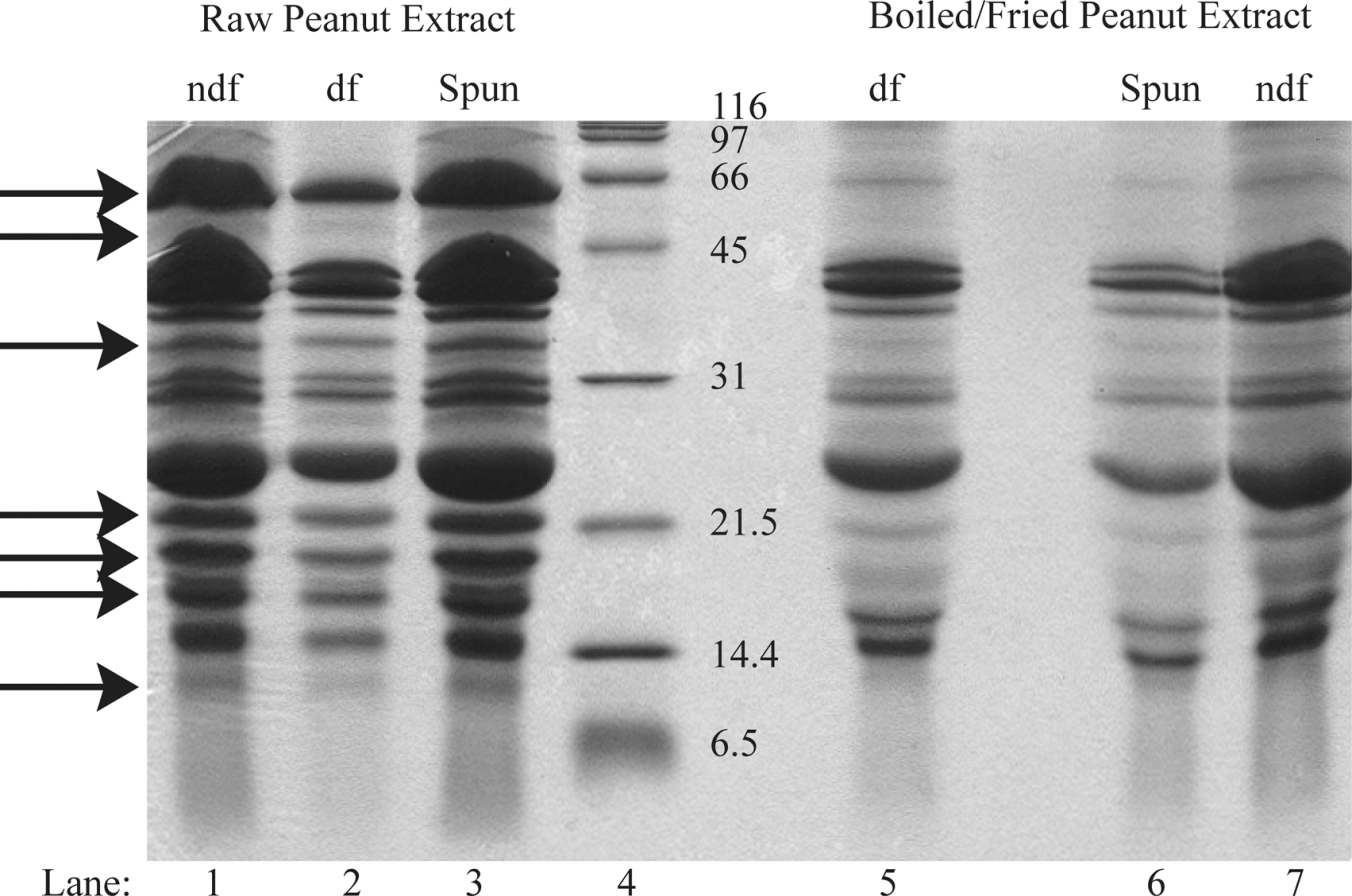
Comparison of chloroform/methanol defatted (df) and non-defatted (ndf), buffer D extracted raw and boiled/fried peanut samples. 50μg protein (as determined using the Bradford assay on extracts prior to centrifugation) was loaded per lane on a 14% reducing SDS-PAGE. Lane labeled “spun” was loaded with non-defatted samples that had been centrifuged for one minute at 15,000 rpm in a benchtop eppendorf centrifuge. Arrows indicate differentially detected proteins.

To determine if defatting changed the protein profile of a peanut extract, a 14% SDS-PAGE gel was run under reducing conditions (Fig 1). Peanut pastes were either defatted (df) or not defatted (ndf) and then extracted. The resulting df and ndf extracts were then either centrifuged at 10,000 x g in a microcentrifuge for 1 min (spun) or not. There was no visible qualitative change in the protein profile of chloroform/methanol defatted extracts (lane 1 vs. lane 2 and lane 7 vs. lane 5). Centrifugation of the ndf boiled/fried sample had an apparent impact on protein yield (lane 6 vs. lane 7). However, for extracts of ndf raw peanuts, centrifugation had no effect on the protein profile (lane 3 vs. lane 1). This likely indicates that extracts from boiled/fried peanuts have more protein aggregates or insoluble proteins than those from raw peanuts.

### Pressure and temperature treatments had little effect on the protein profiles of peanut extracts

**Fig 2:**
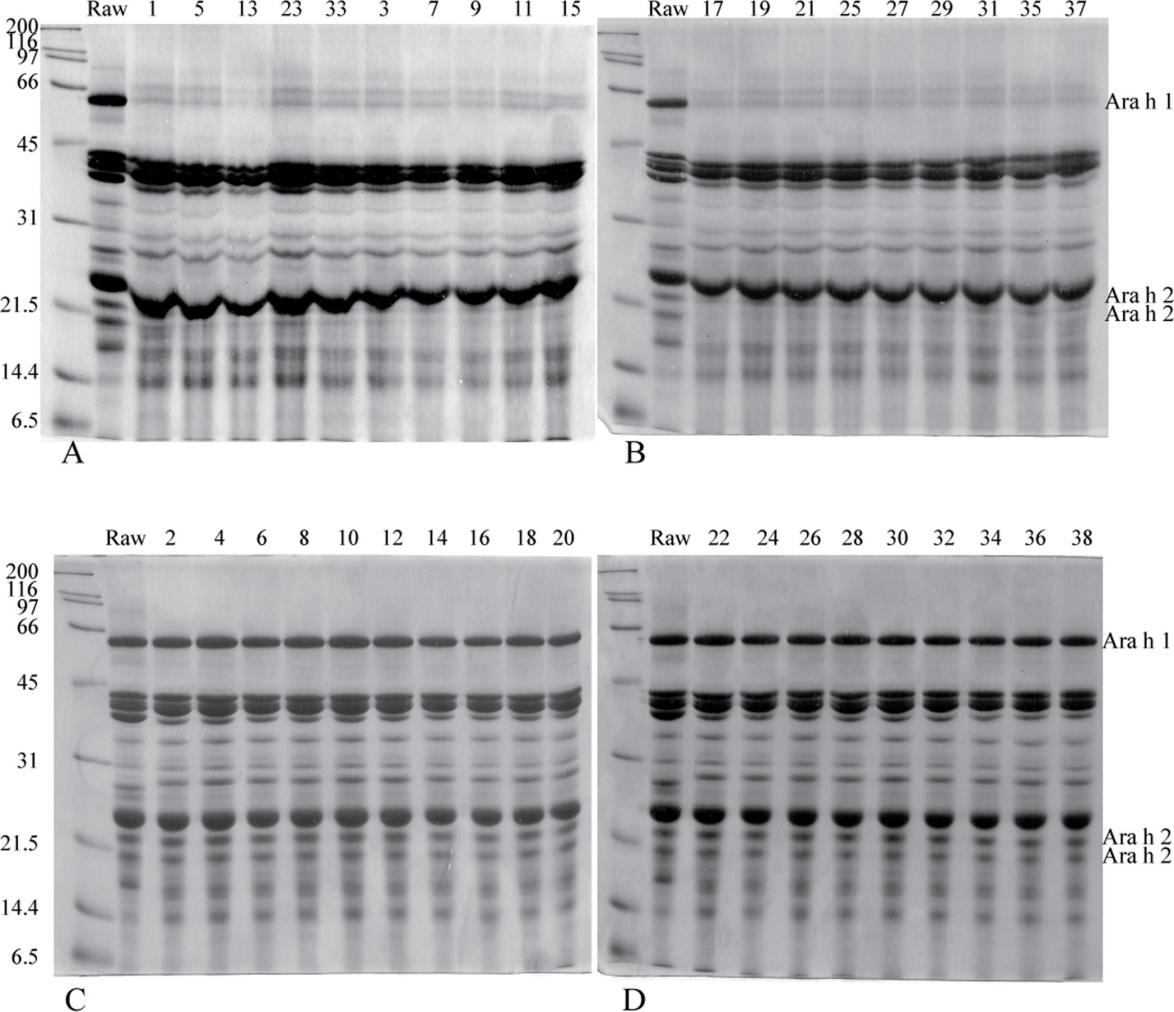
12% reducing SDS-PAGE of all 38 defatted, buffer D extracted peanut extracts. A raw peanut extract (Raw) from defatted peanut was included on each gel as an internal control. Lanes are labeled with extract number (Table 1). Each lane was loaded with 65μg of protein.

Treatment of the peanut samples with combinations of particular pressures and temperatures for specific amounts of time did not appreciably affect the protein profiles of the peanut samples ((Fig 2), see Table 1 for the list of treatments). This was true whether the peanuts were initially treated by boiling/frying (Fig 2A and 2B) or raw (Fig 2C and 2D).

### The boiling/frying procedure changes the SDS-PAGE protein profile of peanut extracts

Although pressure and temperature treatments did not have an effect on the protein profiles of the peanut samples, the boiling/frying step significantly affected the extractable peanut protein profile. In SDS-PAGE analysis of the various peanut extracts it can be seen that boiled/fried samples (Fig 2A and 2B) are missing bands at the molecular weights where known major peanut allergens, Ara h1 (63kDa) and Ara h 2 (a doublet at 20kDa and 18 kDa), have been shown to migrate. To confirm the difference in protein profiles between boiled/fried and raw peanut samples, a higher percentage (14%) gel was run and protein samples were overloaded (Figure A in S1 File). A protein band at 63kDa was significantly decreased in boiled/fried soluble extracts. Since this is the expected MW of Ara h 1, this decrease confirmed that Ara h 1 as an extractable monomer was less abundant in the boiled/fried extracts. The Ara h 2 doublet was also absent or modified such that the peptides did not migrate at their expected MWs in the boiled/fried samples. The band appearing near 200kDa could correspond to Ara h 1 trimers as these have been reported to be present in roasted peanut samples [20,26]. Additionally, protein bands were missing or less abundant around 50kDa, 34kDa, 27kDa, 22kDa, and 16kDa in the boiled/fried peanut samples. Some of these peptides may correspond to other peanut allergens such as Ara h 3, Ara h 6, Ara h 8 or Ara h 9.

### IgE from peanut allergic individuals binds to all extracts

**Fig 3:**
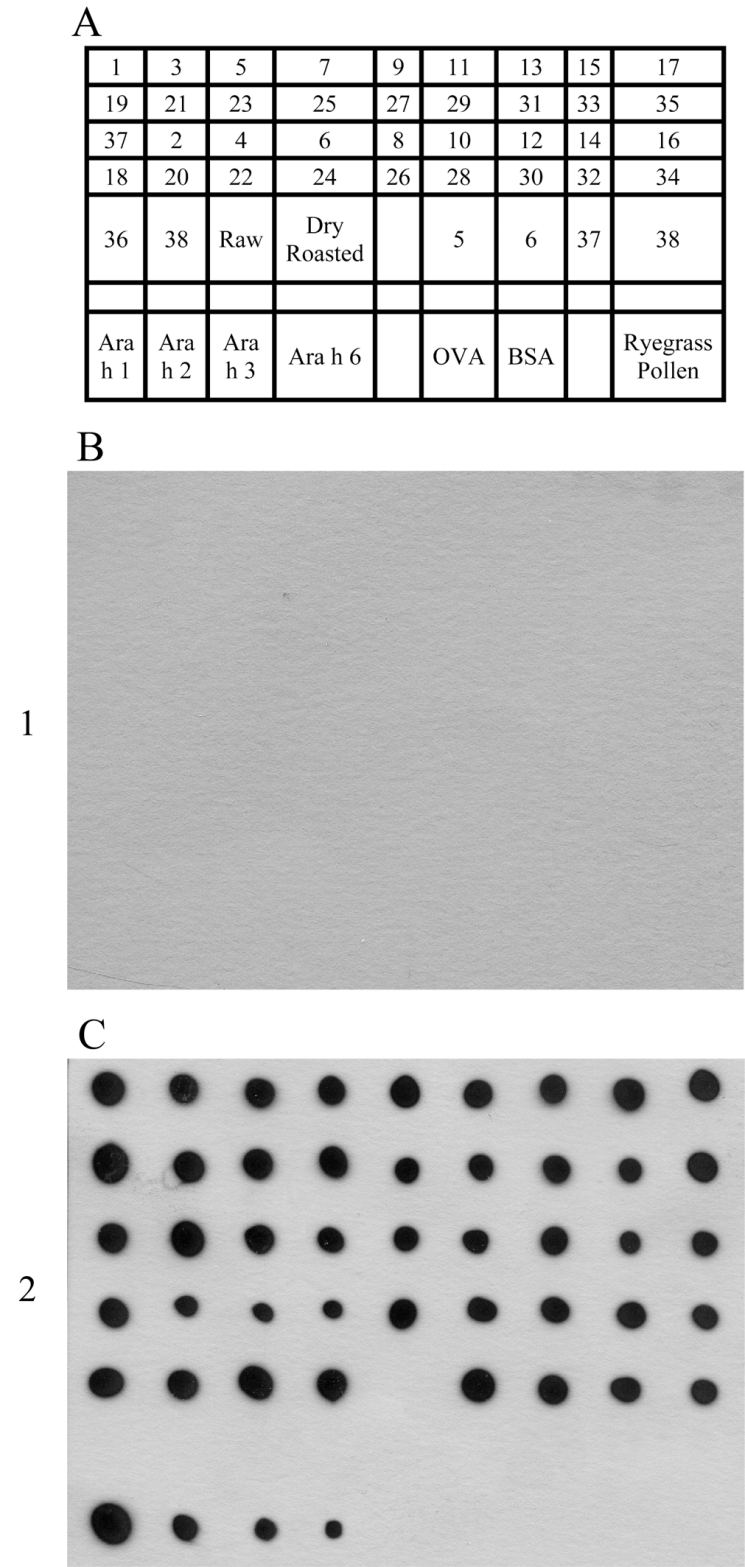
Dot blot of all 38 defatted, buffer D extracted peanut samples (1μg/dot). (A) Dot blot layout. Samples were blotted with serum from an atopic (mold sensitive), non-food allergic patient with presumably no peanut-specific IgE (B) and a peanut allergic individual (C).

**Fig 4:**
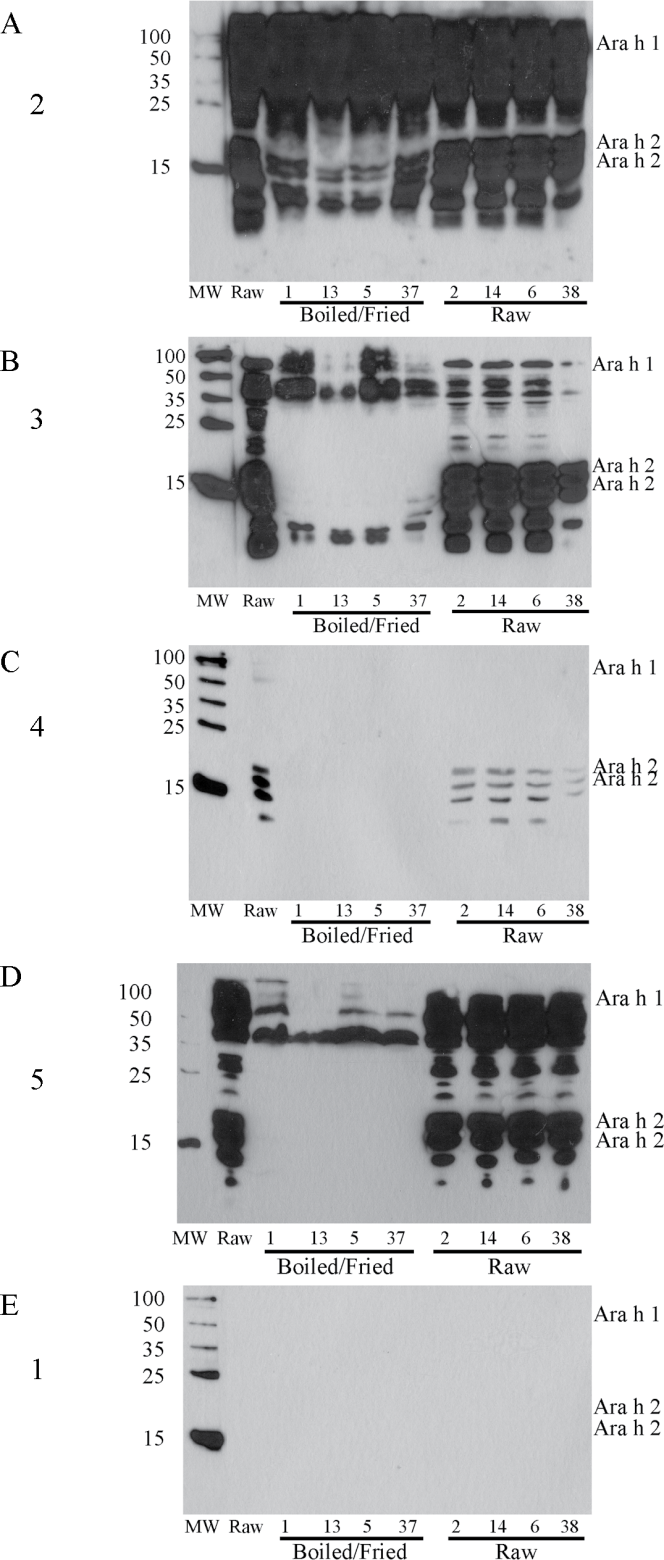
Immunoblots of defatted, buffer D extracted peanut samples (numbers match Table 1) blotted with serum from peanut allergic individuals. Serum was from individuals with a broad IgE repertoire (A, B), individuals with a more limited IgE repertoire (C, D), or an atopic (mold sensitive), non-food allergic patient with presumably no peanut-specific IgE (E). Extracts were run on 14% SDS-PAGE, 50μg per lane, and transferred to 0.22μm NC before blotting.

To determine if the boiling/frying procedure changed overall IgE-reactive epitopes in the peanut proteins, a dot blot (Fig 3) was done using serum from a severely peanut allergic individual (anaphylaxis to trace amounts of peanut per history). While IgE from an atopic control (Fig 3B) did not react with any peanut extracts, IgE from a peanut allergic individual (Fig 3C) reacted with all peanut extracts, but with varying strengths. The peanut allergic serum that was used contained IgE to purified Ara h 1, Ara h 2, Ara h 3, and Ara h 6 (Figs 3C and 4A). This indicates that all of the peanut samples tested have the potential to bind IgE and most likely cause reactions in peanut allergic individuals. However, these experiments cannot predict which proteins the individual is reacting to or if the total quantity of peanut required to elicit a reaction might be altered.

Since serum IgE from a peanut allergic patient bound to all extracts in dot blotting (Fig 3C), immunoblots (Fig 4) were performed on peanut samples to identify specific IgE-reactive proteins present in each extract. Significant differences in binding profiles to allergen or protein can be seen between the soluble extract of boiled/fried and raw samples for all serum samples tested. There is less IgE binding to boiled/fried samples for all serum samples tested (Fig 4A–4D). The most striking difference can be seen for serum from subject 4 (Fig 4C), where no IgE binds the extracts from boiled/fried samples. For serum from the individual with the broadest peanut-specific IgE repertoire (used in the dot-blot Fig 3C), there is significant binding to all extracts tested, both raw and boiled/fried (Fig 4A).

### Ara h 1, Ara h 2 and Ara h 6 proteins in boiled/fried peanut samples are not recognizable by polyclonal allergen-specific antibodies synthesized in chickens

**Fig 5:**
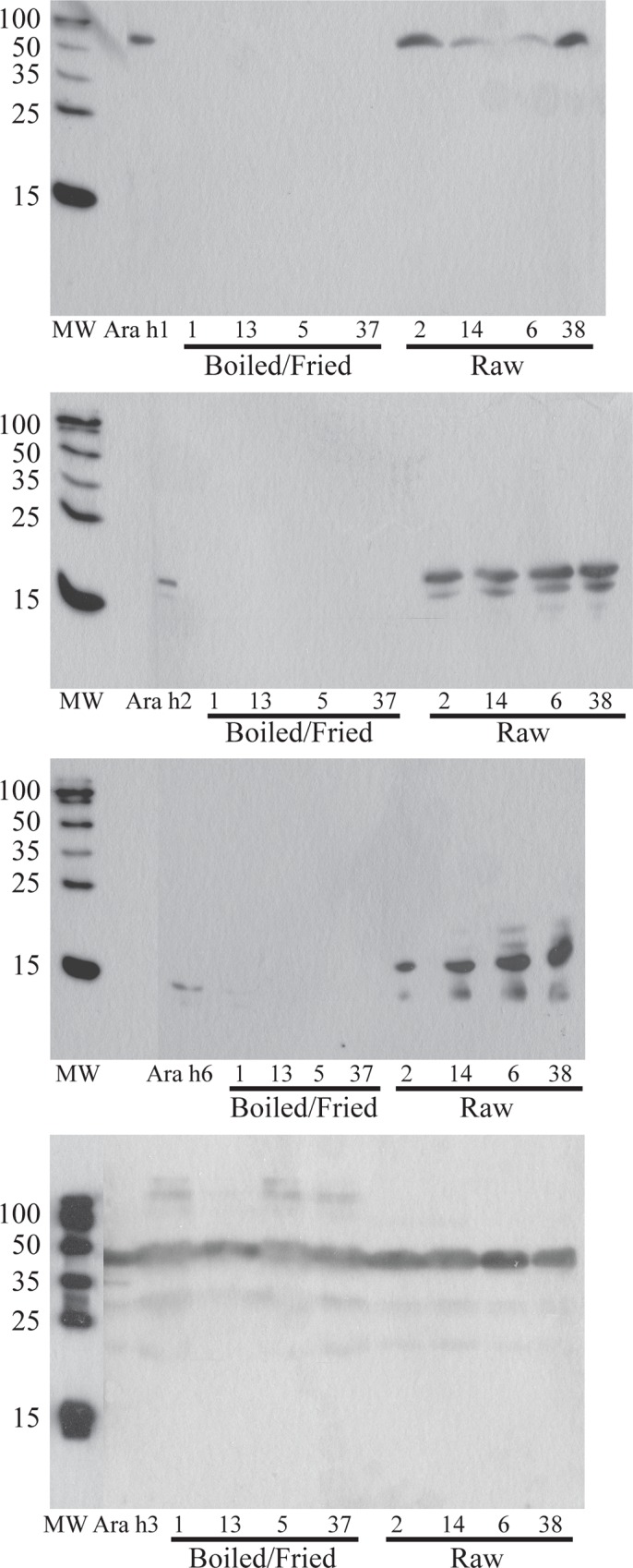
Immunoblots of defatted, buffer D extracted peanut samples (numbers match Table 1) blotted with polyclonal galline anti-Ara antibodies. A) anti-Ara h 1, B) anti-Ara h 2, C) anti-Ara h 6 and D) anti-Ara h 3. Extracts were run on 14% reducing SDS-PAGE, 50μg per lane, and transferred to 0.22μm NC before blotting.

To determine if peanut allergens in boiled/fried peanut samples have lost their IgE-binding epitopes, or are running at unexpected molecular weights, several raw and several boiled/fried peanut samples were immunoblotted with anti-Ara h 1, anti-Ara h 2, anti-Ara h 6 (because of its proposed homology to and cross-reactivity with Ara h 2 [9]), and anti-Ara h 3 (Fig 5). While pronounced binding to Ara h 1, 2 and 6 could be seen in raw peanut samples, no Ara h 1, 2 or 6 could be detected in the boiled/fried peanut samples (Fig 5A–5C). Ara h 3 was detected in all peanut samples tested (Fig 5D). We did not subject solids leftover from extraction to further extraction in differing buffers or further analysis in this study.

### IgE-reactivity to extract from raw peanut is entirely removed by preincubation of human sera samples with extract of boiled/fried peanuts

**Fig 6:**
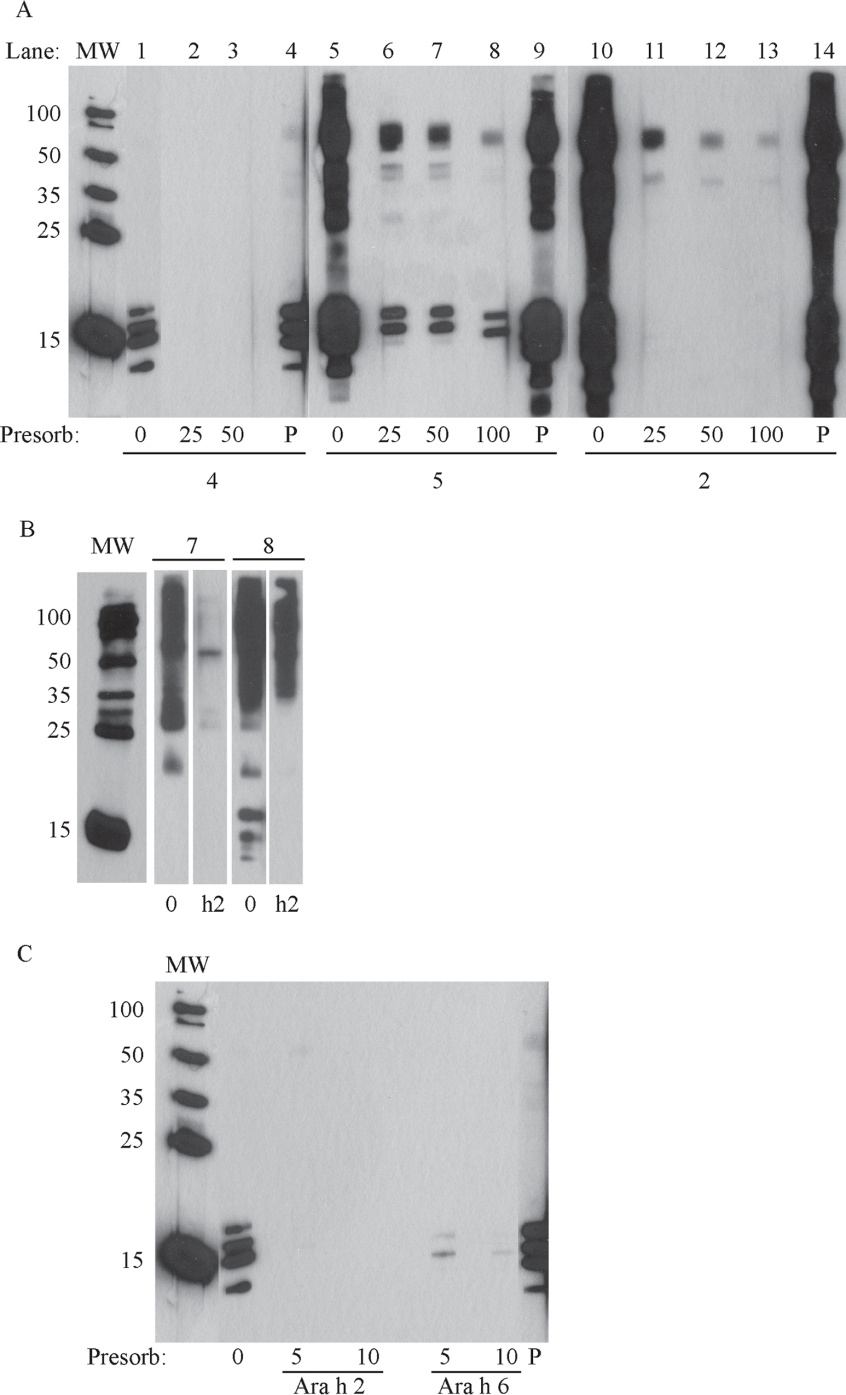
Inhibition immunoblots. A) Inhibition immunoblot of raw peanut (sample 38) with sera from peanut allergic individuals presorbed with boiled/fried peanut (sample 37). B) Inhibition immunoblot of boiled/fried peanut (sample 37) with sera from peanut allergic individuals presorbed with purified Ara h 2 protein (h2) or not presorbed (0). C) Inhibition immunoblot of raw peanut (sample 38) with sera 4 presorbed with purified Ara h 2 (5μg or 10μg) or Ara h 6 (5μg or 10μg). “P” indicates a sample was presorbed with pollen extract.

Next, inhibition immunoblots were performed using serum from individuals with peanut allergy. These blots would confirm that the lack of antibody binding to the peanut allergens Ara h 1, Ara h 2 and Ara h 6 was due to the absence of those proteins in the soluble portion of boiled/fried extracts and not because conformational changes caused by protein gel electrophoresis. Upon inhibition immunoblot of raw peanut samples using sera preincubated with boiled/fried extract, all IgE binding was inhibited for serum from subject 4 (Fig 6A, lanes 2–3). Binding to raw peanut extract was partially inhibited for the two more broadly reactive sera (5 and 2, Fig 6A, lanes 8 and 13). This indicates that nearly all IgE-reactivity to raw peanuts, with the exception of Ara h 1 and/or Ara h 2, depending on the patient, can be removed by proteins found in the boiled/fried extracts, even though IgE binding (for some subjects) to these proteins was not detectable in the boiled/fried sample extracts, upon immunoblot. Additionally, an inhibition immunoblot of the treated sample with sera presorbed with purified Ara h 2, showed that preincubation of some serum samples with purified Ara h 2 removed most reactivity to the boiled/fried sample at multiple MWs in addition to those corresponding to Ara h 2’s expected MW (Fig 6B, subjects 7 and 8). Finally, preincubation of serum from subject 4 with either Ara h 2 or Ara h 6 removed all IgE binding to raw peanut extract (Fig 6C) indicating that the IgE in subject 4’s serum recognizes proteins that contain epitopes similar to Ara h 2 and 6.

## Discussion

The majority of the Ara h 1 and Ara h 2 proteins do not migrate at their expected molecular weights in soluble protein extracts obtained from samples that were boiled and then fried (Figs 1 and 2). Some residual Ara h1 and Ara h 2 proteins migrate at their expected MWs but are dramatically reduced relative to raw peanut extract. This indicates that the majority of Ara h 1 and Ara h 2 have been chemically and/or structurally altered, destroyed entirely or rendered insoluble by the boiling/frying procedure.Immunoblotting experiments and functional assays (basophil activation testing, Figure B in S1 File) were undertaken. IgE from peanut allergic individuals, whose sera have broad peanut reactivity, bound proteins in extracts from both boiled/fried and raw peanut samples (Fig 3C (dot blotting), Fig 4A, 4B and 4D (immunoblotting)). However, IgE from one subject did not bind proteins in boiled/fried samples (Fig 4C). Additionally, polyclonal antibodies to Ara h 1, Ara h 2, and Ara h 6 did not bind proteins in soluble extracts from boiled/fried peanuts (Fig 5). Despite this evidence of lower levels of soluble Ara h 1 and Ara h 2 in boiled/fried sample extracts, incubation of the more restricted serum (subject 4) with the boiled/fried peanut extract inhibited all reactivity to raw peanut extract and incubation with two other more broadly reactive sera (subjects 2, 5) partially inhibited binding to raw peanut extract (Fig 6A). IgE binding is not equivalent to functional activity, thus CD63/203c FACS detection of basophil activation was used to determine if proteins extracted from boiled/fried peanuts could trigger basophil activation. Both raw and boiled/fried peanuts activated basophils loaded with serum from peanut allergic individuals (Figure B in S1 File). These results indicate that although there is a decreased amount of some peanut allergens or the allergens are altered in boiled/fried peanuts, peanuts processed in this manner remain likely to trigger allergic responses from most peanut allergic individuals.

Impaired protein solubility or chemical/structural changes to the proteins could explain the discrepancies in results from experiments where proteins are denatured (SDS-PAGE and direct immunoblotting) compared to those where proteins are folded (as in inhibition immunoblotting or basophil activation testing). It has been shown that roasting, boiling or frying peanuts can decrease the solubility of the extracted proteins [25,31]. Thus, the proteins which seem to be missing from boiled/fried peanut extracts likely were insoluble or aggregated such that they could not be extracted into solution from the processed samples or, perhaps, were unable to enter a polyacrylamide resolving gel. However, proteins with those properties would be available to bind to IgE in a solution, such as those used in inhibition immunoblotting or basophil activation testing. Structural changes in the protein caused by boiling and/or frying could also explain the discrepancy. When proteins are in their native, folded form rather than denatured, IgE molecules may more readily recognize common chemical/structural modifications caused by boiling/frying [39]. However, the effect of heat processing on at least one of the allergens, Ara h 1, has been examined. Although purified, native Ara h 1 undergoes heat-induced denaturation between 80°C—90°C, the IgE-binding properties of Ara h 1 are not affected [40]. These studies were done under dry heat conditions and tested temperatures from 50°C to 200°C. However, others have also shown that boiling purified Ara h 2 or Ara h 1 has no effect on their electrophoretic or blotting profiles [27]. In the context of the entire peanut matrix, boiling or frying has been shown to aggregate Ara h 1 and Ara h 3 altering their electrophoretic mobility [36].

Other additional explanations help reconcile why boiled/fried peanut extract inhibits some IgE binding to raw peanut extract and activates basophils loaded with serum from peanut allergic individuals, despite the apparent absence of major allergens in SDS-PAGE and direct immunoblot with polyclonal antibodies from chickens as well as some subject serum samples. The first is the presence of small quantities of Ara h 1 and Ara h 2 in the extract. The quantity of protein present may be undetectable by Coomassie blue staining (limit of detection from 0.1 to 1μg/band, Bio-Rad R-250 product information) but adequate to bind IgE in solution and thereby inhibit binding to proteins in the raw samples. Alternatively, other proteins in the boiled/fried extract may bind serum IgE that would normally recognize Ara h 1 or Ara h 2. This is supported by the evidence that purified Ara h 2 could inhibit IgE binding to a wide array of peanut proteins (Fig 6B) and recent work by others which demonstrated extensive cross-reactivity between Ara h 1 and Ara h 3 with Ara h 2 [38]. Furthermore, basophil activation testing may have a higher level of sensitivity and specificity compared to immunoblot [41].

Other studies have sought to identify processing methods which reliably decrease the allergens in peanuts. Frying and boiling have previously been studied as separate processes to alter the allergenicity of peanuts [20,27,36]. In these studies, as well as in our work, IgE from most peanut allergic individuals bound to peanut proteins in all samples, albeit to a somewhat lesser extent in samples from peanuts which had been boiled or fried. These studies support our finding that boiling, even when followed by frying, decreases the presence of Ara h 2, Ara h 1 and other low MW proteins in soluble peanut extracts [20,27,36]. Although it has been shown that roasting peanuts decreases the solubility of proteins [25,28], paradoxically more IgE binds to proteins from roasted peanuts than to those from raw peanuts [26]. This is perhaps due to Maillard reactions generating protein/sugar complexes [21,22]. The differences in temperature among roasting (~140°C), boiling (100°C), and frying (~175°C) may explain the different effects on proteins and IgE binding. It is also possible the differences are due to the fact that roasting occurs under dry conditions while boiling and frying involve liquid. When Mondoulet, et al examined the water in which the peanuts were boiled [27], they found Ara h 2 in the water. We did not examine the water in which the peanuts were boiled. However, if the allergen is lost into the water, this would enable processors to literally pour off the allergen before using the processed peanuts in baked goods or other products. Notably, in both the work presented herein and that done by Mondoulet, et. al., the peanuts were boiled as full kernels. A more complete removal of Ara h 2 might be attained if peanut powders were boiled rather than the full kernels. In fact, Turner et al [33] used defatted flours from boiled peanuts in similar experiments showing that many IgE-reactive proteins with MW of 28kDa or less were recovered in the water.

We must emphasize that exposure to minute quantities of allergen can cause severe reactions in some peanut allergic individuals. In one carefully controlled study, the minimal dose to elicit a reaction to peanut was experimentally determined as 100μg of peanut protein confirming a threshold value that had been previously reported [42,43]. In another study, the minimal dose to elicit symptoms was found to be 10mg of peanut protein [44]. Thus, if even small amounts of allergens remain in the boiled/fried peanuts, the peanuts cannot be considered hypoallergenic.

We have shown that extracts from boiled/fried peanuts can activate basophils loaded with IgE from two peanut allergic individuals and can remove all or most IgE binding to raw peanut extracts. This indicates that some IgE-binding proteins still are present in the extract, and thus the boiled/fried peanuts are not hypoallergenic. Although the data presented herein show that the boiled/fried peanut is not hypoallergenic, the decrease in Ara h 2 seen when peanuts were boiled/fried is significant since Ara h 2 is felt to be the most clinically important allergen in many populations [7,45,46]. Many peanut allergic individuals have IgE to Ara h 2 [45–47], and it has been shown that IgE from peanut allergic individuals have a higher apparent affinity for Ara h 2 than Ara h 1 [27]. Thus it may be that Ara h 2 is an important sensitizing protein. If so, peanuts with decreased Ara h 2 or altered Ara h 2 may be less likely to sensitize individuals.

## Supporting Information

S1 File **Figure A.** Overloaded gel of raw and boiled/fried peanut extracts (numbers match Table 1). 14% SDS-PAGE with samples loaded at 100μg (as determined using the Bradford assay on extracts) per lane with reducing sample buffer. The major peanut allergens, Ara h 1 and Ara h 2, are labeled. **Figure B.** Dot plots from modified basophil activation testing. Basophils were loaded with IgE from a peanut allergic individual and than challenged with PBS (Unstim), bovine serum albumin (BSA), the peptide fMLP (fMLP), or various concentrations of raw or boiled/fried peanut extract. A) Basophils loaded with serum from subject 3. B) Basophils loaded with serum from subject 6.
(DOCX)
