# Supplementary material for: Boiling and Frying Peanuts Decreases Soluble Peanut (Arachis Hypogaea) Allergens Ara h 1 and Ara h 2 But Does Not Generate Hypoallergenic Peanuts
Source: PLoS One. 2016 Jun 16;11(6):e0157849. doi: 10.1371/journal.pone.0157849 (PMC4911009; doi:10.1371/journal.pone.0157849)
Supplement: S1 File — Figure A. Overloaded gel of raw and boiled/fried peanut extracts (numbers match Table 1). 14% SDS-PAGE with samples loaded at 100μg (as determined using the Bradford assay on extracts) per lane with reducing sample buffer. The major peanut allergens, Ara h 1 and Ara h 2, are labeled. Figure B. Dot plots from modified basophil activation testing. Basophils were loaded with IgE from a peanut allergic individual and than challenged with PBS (Unstim), bovine serum albumin (BSA), the peptide fMLP (fMLP), or various concentrations of raw or boiled/fried peanut extract. A) Basophils loaded with serum from subject 3. B) Basophils loaded with serum from subject 6. (DOCX) [file pone.0157849.s001.docx]

**S1. Supporting Information**

Boiling and Frying Peanuts Decreases Soluble Peanut (Arachis Hypogaea) Allergens Ara h 1 and Ara h 2 but does not Generate Hypoallergenic Peanuts

Sarah S. Comstock, Soheila J. Maleki, Suzanne S. Teuber

*Both treated and raw peanut samples activate basophils loaded with IgE from peanut allergic individuals.*

Since IgE binding cannot predict functional activity, CD63/203c flow cytometric detection of basophil activation was used to determine if proteins extracted from boiled/fried peanuts could trigger basophil activation. To test if the differences in IgE binding had potential *in vivo* significance, a basophil activation test was run. Upon activation human basophils (CD203c^+^) upregulate CD63 expression at their cell surface, thus events in the upper right quadrant of the dot plot are activated basophils. For basophils loaded with serum IgE from one peanut allergic individual, a larger quantity of boiled/fried peanut sample than raw peanut sample was needed to activate basophils (Fig 2 in S1 file) as seen by the increased number of events in the upper right quadrant of the dot blots for raw peanut incubations. However, for basophils loaded with serum IgE from another peanut allergic individual, both samples activated basophils when provided at the lowest quantity tested, 1µg (Fig 2B in S1 file) as seen by the large number of events in the upper right quadrant of the dot blots for both boiled/fried and raw peanut incubations. This indicates that both raw and boiled/fried peanuts are likely to cause reactions in individuals with peanut allergy.

### Basophil Activation Testing Methods

Basophil activation testing (BAT) and flow cytometry staining was done as previously described (*1*). Briefly, basophils were enriched from whole blood collected from non-food allergic, atopic human donors. IgE was stripped from the basophils using a lactic acid buffer [13.4 mM lactic acid, 140 mM NaCl, 5mM KCl; pH 3.9]. Cells were then loaded with IgE from peanut allergic individuals by incubating cells with serum in the presence of 4mM EDTA and 10µg/ml heparin in a 37°C water bath for 90 minutes. Allergen challenge consisted of incubating cells with peanut samples for exactly 15 min at 37°C. Cells were then stained for flow cytometry and analyzed using a FACScan (Becton Dickinson, Franklin Lakes, NJ).

1. Wallowitz, M.; Chen R.; Tzen, J.; Teuber S. Ses i 6, the sesame 11S globulin, can activate basophils and shows cross-reactivity with walnut *in vitro*. *Clin Exp Allergy* **2007**, 37, 6, 929-38.

**Fig A in S1 file**:

Overloaded gel of raw and boiled/fried peanut extracts (numbers match Table 1). 14% SDS-PAGE with samples loaded at 100µg (as determined using the Bradford assay on extracts) per lane with reducing sample buffer. The major peanut allergens, Ara h 1 and Ara h 2, are labeled.

**Fig A in S1 file**.


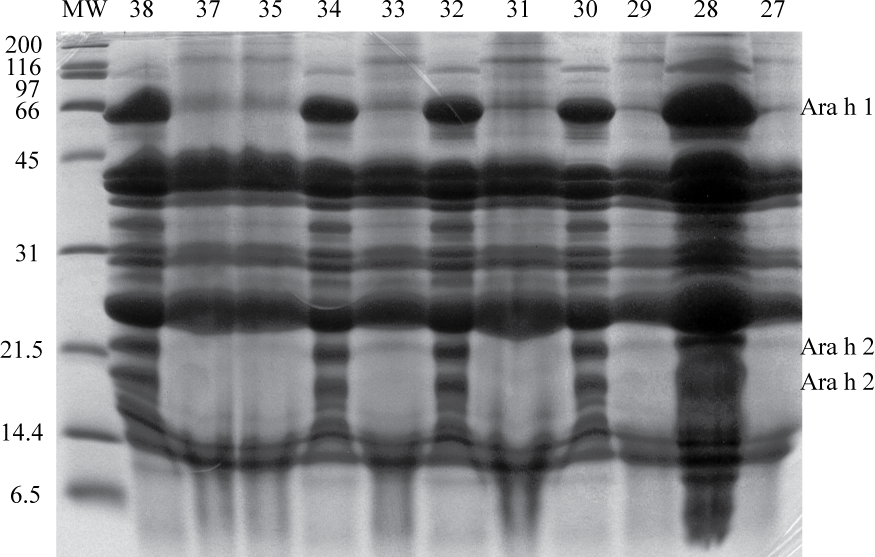


**Fig B in S1 file**: Dot plots from modified basophil activation testing. Basophils were loaded with IgE from a peanut allergic individual and than challenged with PBS (Unstim), bovine serum albumin (BSA), the peptide fMLP (fMLP), or various concentrations of raw or boiled/fried peanut extract. A) Basophils loaded with serum from subject 3. B) Basophils loaded with serum from subject 6.

**Fig B in S1 file.**
